# Retraction statement: Long noncoding RNA MALAT1 promotes the stemness of esophageal squamous cell carcinoma by enhancing YAP transcriptional activity. FEBS Open Bio, 9: 1392–1402.

**DOI:** 10.1002/2211-5463.13706

**Published:** 2023-10-04

## Abstract

https://doi.org/10.1002/2211‐5463.12676

The above article, published online on 22 May 2019 in Wiley Online Library (wileyonlinelibrary.com), has been retracted by agreement between the Editor‐in‐Chief Miguel A. De la Rosa, FEBS Press, and John Wiley and Sons Ltd. The retraction has been agreed following an investigation into concerns raised by a third party, which revealed inappropriate duplications between this and previously published articles [1–3]. Thus, the editors consider the conclusions of this manuscript substantially compromised.

[1] RETRACTED: Wu, H., He, Y., Chen, H., Liu, Y., Wei, B., Chen, G., Lin, H. and Lin, H. (2019), Retracted: LncRNA THOR increases osteosarcoma cell stemness and migration by enhancing SOX9 mRNA stability. FEBS Open Bio, 9: 781–790. https://doi.org/10.1002/2211‐5463.12620

[2] Xiao, Y., Pan, J., Geng, Q. and Wang, G. (2019), LncRNA MALAT1 increases the stemness of gastric cancer cells via enhancing SOX2 mRNA stability. FEBS Open Bio, 9: 1212–1222. https://doi.org/10.1002/2211‐5463.12649

[3] Hou, H, Yu, X, Cong, P, Zhou, Y, Xu, Y, Jiang, Y. (2019). Six2 promotes non–small cell lung cancer cell stemness via transcriptionally and epigenetically regulating E‐cadherin. Cell Prolif.; 52:e12617. https://doi.org/10.1111/cpr.12617

https://doi.org/10.1002/2211‐5463.12676

The above article, published online on 22 May 2019 in Wiley Online Library (wileyonlinelibrary.com), has been retracted by agreement between the Editor‐in‐Chief Miguel A. De la Rosa, FEBS Press, and John Wiley and Sons Ltd. The retraction has been agreed following an investigation into concerns raised by a third party, which revealed inappropriate duplications between this and previously published articles [1–3]. Thus, the editors consider the conclusions of this manuscript substantially compromised.

[1] RETRACTED: Wu, H., He, Y., Chen, H., Liu, Y., Wei, B., Chen, G., Lin, H. and Lin, H. (2019), Retracted: LncRNA THOR increases osteosarcoma cell stemness and migration by enhancing SOX9 mRNA stability. FEBS Open Bio, 9: 781–790. https://doi.org/10.1002/2211‐5463.12620

[2] Xiao, Y., Pan, J., Geng, Q. and Wang, G. (2019), LncRNA MALAT1 increases the stemness of gastric cancer cells via enhancing SOX2 mRNA stability. FEBS Open Bio, 9: 1212–1222. https://doi.org/10.1002/2211‐5463.12649

[3] Hou, H, Yu, X, Cong, P, Zhou, Y, Xu, Y, Jiang, Y. (2019). Six2 promotes non–small cell lung cancer cell stemness via transcriptionally and epigenetically regulating E‐cadherin. Cell Prolif.; 52:e12617. https://doi.org/10.1111/cpr.12617

